# A fatal rhino-orbit-cerebral mucormycosis infection aggravated by coronavirus disease-2019

**DOI:** 10.1590/0037-8682-0666-2021

**Published:** 2022-04-08

**Authors:** Akif İşlek, Sadullah Şimşek

**Affiliations:** 1 Acıbadem Eskişehir Hospital, Otolaryngology-Head & Neck Surgery Clinic, Eskişehir, Turkey.; 2 Dicle University, Department of Radiology, Diyarbakır, Turkey.

Rhino-orbit-cerebral mucormycosis is an opportunistic infection caused by *Rhizopus* spp., *Mucor* spp., and other mucormycosis with high morbidity and mortality^1^. The main risk factors for mucormycosis include uncontrolled diabetes mellitus, diabetic ketoacidosis, other forms of metabolic acidosis, corticosteroid treatment, organ or bone marrow transplantation, neutropenia, trauma and burns, malignant hematologic disorders, and iron overload^2^.

**FIGURE 1:**
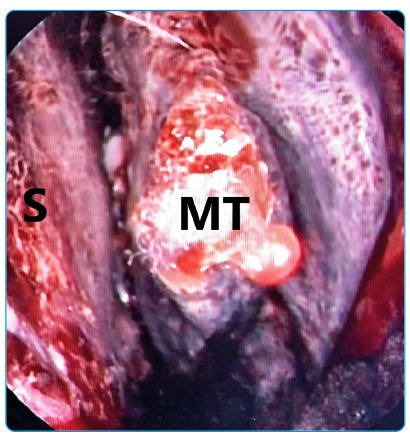
Purple-black discoloration on the left nasal endoscopy. **S:** septum; **MT:** middle turbinate.

**FIGURE 2:**
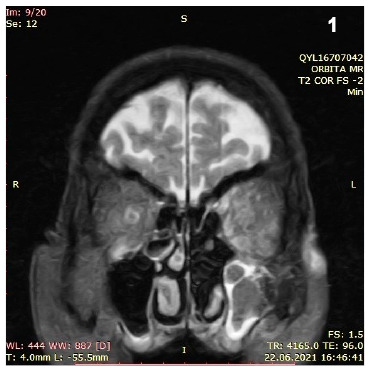
T2 orbital MR, coronal slice shows complete left maxillary sinus involvement.

**FIGURE 3:**
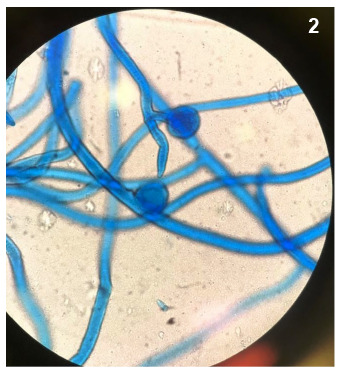
Lactophenol cotton blue staining, the broad aseptate hyphae with the extension of the columella into the sporangium (x100).

A 75-year-old man was hospitalized for COVID-19 pneumonia on June 14, 2021. The patient was referred to the ear-nose-throat clinic due to vision loss in the left eye and persistent maxillary toothache on the left side on day 8 of hospitalization. Left nasal endoscopy detected purple-black discoloration and paresthesia (Figure 1). Magnetic resonance imaging revealed a left maxillary sinus and orbital involvement (Figure 2). Aseptate hyphae and sporangium structures of *Rhizopus sp.* were detected in tissue samples (Figure 3). Therefore, amphotericin (5 mg/kg/day) treatment was initiated. Endoscopic extended surgical debridement was performed on day 9. Diffuse necrosis was observed in the left nasal cavity during endoscopic surgery. Three days after the operation, the patient was transferred to the intensive care unit (ICU) for respiratory distress. The patient died on day 5 (July 1, 2021) of the ICU follow-up.

Although mucormycosis often develops in diabetic patients, Covid-19 disease causes lymphopenia and T-cell dysfunction^3^, which facilitates coinfection in this case.
